# Implementation of a co-designed physical activity program for older adults: positive impact when delivered at scale

**DOI:** 10.1186/s12889-018-6210-2

**Published:** 2018-11-23

**Authors:** Heather McKay, Lindsay Nettlefold, Adrian Bauman, Christa Hoy, Samantha M. Gray, Erica Lau, Joanie Sims-Gould

**Affiliations:** 1 Centre for Hip Health and Mobility, Vancouver Coastal Health Research Centre, 7th Floor Robert H.N. Ho Research Centre, 795-2635 Laurel St, Vancouver, BC V5Z 1M9 Canada; 2 Department of Family Practice, University of British Columbia, 3rd Floor David Strangway Building, 5950 University Boulevard, Vancouver, BC V6T 1Z3 Canada; 3 Sydney School of Public Health, University of Sydney, Charles Perkins Centre, Building D17, Camperdown, NSW 2006 Australia

**Keywords:** Physical activity, Mobility, Social connectedness, Seniors, Older adults, Implementation science, Impact evaluation, Scale up

## Abstract

**Background:**

Despite known health benefits of physical activity (PA), older adults remain among the least physically active age group globally with 30–60% not meeting guidelines. In Canada, 87% do not meet recommended guidelines. To influence population health, interventions that are effective in small trials must be disseminated at scale. Despite evidence for efficacy, few PA interventions are scaled up to reach the wider community. In 2015, British Columbia (BC) Ministry of Health released a PA strategy where older adults were identified as a priority. In partnership with the Ministry, the Active Aging Research Team co-created a health promotion program called Choose to Move (CTM). CTM will be implemented in three phases at increasingly greater scale across BC. The objective of this study is to evaluate the effectiveness of CTM during Phase I (pilot) and Phase II (initial scale up) on PA, mobility, and social connectedness among older adults in BC, Canada.

**Methods:**

We used a type 2 hybrid effectiveness-implementation study design, and herein focus on effectiveness. The implementation evaluation will be published as a companion paper elsewhere. Two community delivery partner organizations delivered 56 CTM programs in 26 large and small urban locations across BC. Outcome measurement occurred at 0 (baseline), 3 (mid-intervention) and 6 (post-intervention) months. We collected survey data from all participants (*n* = 458; province-wide) and also conducted a subset evaluation (*n* = 209).

**Results:**

PA increased significantly during the active intervention phase (baseline-3 months) in younger (60–74 yrs.; + 1.6 days/week; *p* < 0.001) and older (≥75 yrs.; + 1.0 days/week; p < 0.001) participants. The increase was sustained at 6 months in younger participants only, who remained significantly more active than at baseline (+ 1.4 days/week; p < 0.001). Social exclusion indicators declined significantly in the younger group. Mobility and strength improved significantly at 3 months in the younger group, and in both groups at 6 months.

**Conclusions:**

CTM adopted central tenets of implementation science that consider the complicated systems where interventions are delivered to improve public health. In this iteration of CTM we demonstrate that a partner-based health promotion intervention can be effectively implemented across settings to enhance PA, mobility and social connectedness in older adults.

**Electronic supplementary material:**

The online version of this article (10.1186/s12889-018-6210-2) contains supplementary material, which is available to authorized users.

## Background

On a global scale the population segment of those over age 65 is projected to reach 2 billion by 2050 [1]. In Canada, there are now more older adults (≥65 y) than children (≤14 y) [2], and the proportion of seniors is projected to exceed 30% by the year 2050 [3]. Maintaining mobility is “the best guarantee of retaining independence and being able to cope” [4]. Limited mobility predicts disease, disability and mortality [5–11] and is a risk factor for social isolation, cognitive impairment and admission into residential care [7, 12, 13]. Importantly, physical activity (PA) and mobility are inextricably linked [1, 4]. Thus, strategies to enhance PA are important to reduce the risk of chronic disease and preserve older adults’ independence [1]. Despite the clear benefits of PA, older adults are among the least physically active age group globally; 30–60% do not meet PA guidelines [14]. In Canada, 87% of older adults fail to meet PA guidelines [15].

As the emotional and financial burden of chronic disease continues to escalate, policy makers seek evidence as to whether interventions that effectively promoted health on a small scale can be disseminated on a broader scale – while retaining their effectiveness. Indeed the focus on populations rather than individuals is the central tenet of health promotion [16]. However, evidence from PA interventions to support effective implementation at scale is almost non-existent [17, 18]. More specifically, despite substantial evidence to support the need for broadly disseminated PA interventions for older adults, only two interventions targeting older adults were scaled-up [17]. This has been referred to as the ‘know-do’ gap [19] *–* a gap that needs to be bridged if we are to improve health through PA at the population level*.*

Participatory methods [20] and a co-creation approach [21] may be key to achieving sustained action and ultimately population level impact [22]. However, those in public health and well-intentioned academics often fail to engage end-users (i.e. those who will deliver the intervention and those in receipt of it) to design scalable public health interventions [23]. Thus, British Columbia (BC) Ministry of Health, and a host of community partners from across BC (BC Physical Activity Leadership Table), co-designed a PA strategy and action plan called Active People, Active Places (released in 2015) [24]. Older adults were identified as a population of focus and a priority area for action. As a next step Ministry of Health convened an Older Adult Action Planning Committee comprised of academic, NGO, not for profit and health authority representatives from across BC. The committee identified four central tenets of a health promotion intervention for older adults. Adopting these central tenets and based on existing evidence, the *Active Aging Research Team* (AART; comprised of researchers and highly qualified staff at University of British Columbia, Canada) designed Choose to Move (CTM). AART also convened a CTM project team (project team) to support delivery of CTM. The project team comprised of two principal investigators (HM, JSG), international research collaborators, a program manager, and several research assistants to support day-to-day operation and program evaluation. With delivery partners, the project team implemented CTM in three phases at increasingly greater scale across BC.

Therefore, the objective of this study is to evaluate the effectiveness of CTM during Phase I (pilot) and Phase II (initial scale up). Targeted outcomes are PA, mobility, social connectedness and mental health measures (loneliness and happiness) among older adults (≥ 60 years) in BC, Canada.

## Methods

**Fig. 1:**
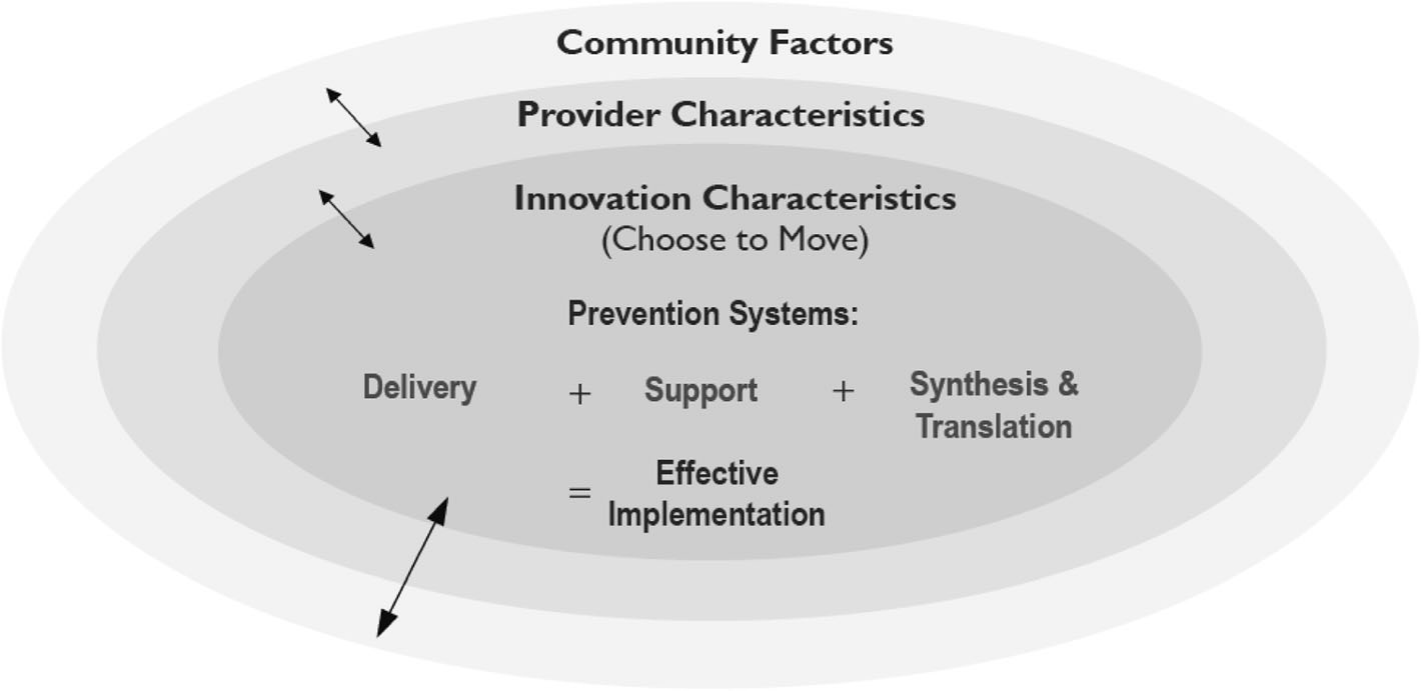
We present the framework for effective implementation. Implementation of the innovation (our intervention - Choose to Move) is guided by the Prevention Delivery System and its organizational capacity, the Prevention Support System and the Prevention Systhesis & Translation System. Together these three elements are deemed essential for effective implementation. Further, these critical components are embedded within a larger context of provider characteristics and community factors (outer rings). Interactions between all components in the outer and central rings are illustrated by bidirectional arrows. Reproduced with permission from the authors [25]

Details of the conceptual frameworks for implementation and evaluation, guiding principles, the intervention (CTM) and evaluation methods that guide our work were described at length in a previous publication [25]. Briefly, the project team adopted core elements of Wandersman’s Interactive Systems Framework [26] and Durlak and DuPre’s ecologic framework [25, 27]. These frameworks incorporate elements of research-to-practice and community-centred implementation models to accommodate the perspectives of a range of stakeholders (Fig. 1).

### Study design and setting

We used a type 2 hybrid effectiveness-implementation study design [28] to evaluate CTM. Type 2 hybrid trials are characterized by two co-primary aims that evaluate effectiveness of the intervention and its implementation. Implementation evaluation encompasses outcomes such as feasibility and potential utility of implementation strategies (e.g., committed delivery partners and phased/tailored approaches for delivery) [25]. However, the focus of this study is on the effectiveness of CTM**.**

**Fig. 2:**
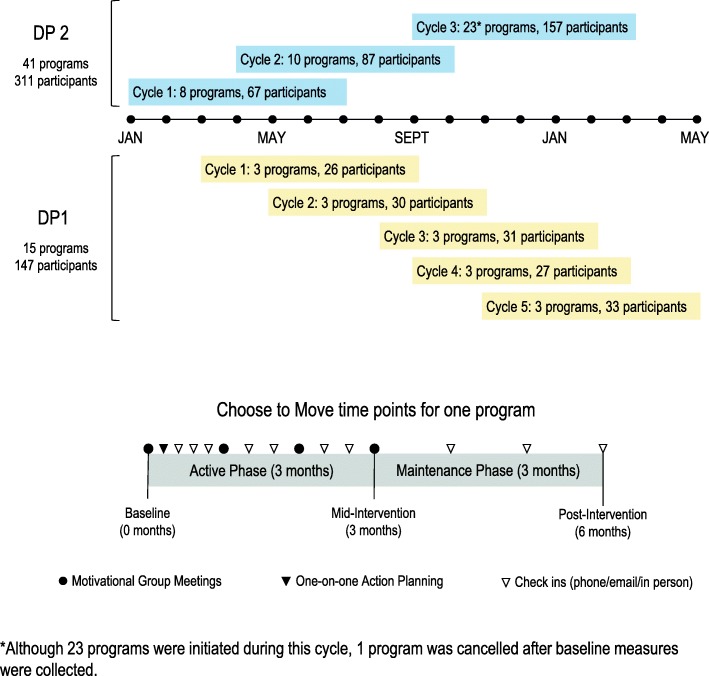
We present the timeline and illustrate the phased delivery and key program components across the time frame of our study

Measurement occurred at 0 (baseline), 3 (mid-intervention) and 6 (post-intervention) months (Fig. 2). CTM (described below) was delivered by two community delivery partner organizations who together had reach to large and small urban communities across BC. Young Men’s Christian Association (YMCA; DP1) is a charitable organization that serves Greater Vancouver with the goal of “helping families thrive” through health promoting activities. British Columbia Recreation and Parks Association (BCRPA; DP2) is a not for profit organization “dedicated to enhancing quality of life in [British Columbia]” with extensive reach to fitness leaders, recreation centres and provincial level programs that serve all regions of BC. Together partner organization affiliates delivered 56 CTM programs between January 2016 and May 2017 in 26 community centres or other facilities using a phased approach (DP1 delivered CTM in 3 sites in Greater Vancouver; DP2 delivered CTM in 23 sites across different regions of BC (Fig. 2).

### Participants

Of 534 CTM participants, 458 consented to participate in the evaluation (86%). Individuals eligible to enrol in CTM were ≥ 60 years, English speaking, and physically inactive (self-reported < 150 min/week of PA) with no contra-indications to PA participation (Physical Activity Readiness-Questionnaire+ [29] or physician clearance). To be eligible at DP1 sites, participants had one or more chronic health conditions. Although many older adult participants presented with a chronic disease at DP2 sites, this was not a strict eligibility criterion. Recruitment strategies varied by site and included local promotions (e.g., program guides, posters, and information sessions), media advertisements (e.g., newspaper, radio, and social media) and word of mouth. The University of British Columbia and Simon Fraser University Clinical Research Ethics Boards (H15–02522 (UBC) and 22,015 s0614 (SFU)) approved all study procedures.

### Choose to move

Co-design of the PA intervention for older adults emerged from the provincial ‘older adult action committee’ comprised of approximately 15 government, NGO, not-for-profit, academic, and health authority stakeholders from across British Columbia. This committee participated in a series of four, three hour in-person workshops (JSG lead). AART conducted a review of the literature to guide committee discussions based on evidence. From these workshops four central tenets of a PA intervention for older adults emerged; 1) focus on enhanced PA, mobility, and social connectedness as key outcomes, 2) include active travel training as one component, 3) engage *physically inactive* older adults (those engaged in < 150 min of moderate-to-vigorous PA/week), and 4) adopt an evidence, choice-based and scalable health promotion intervention that could be adapted to setting, physical capacity and interests.

These central tenets and the available evidence on effective PA interventions for older adults underpinned design of the Choose to Move (CTM) intervention. Specifically, Community Health Activities Model Program for Seniors (CHAMPS) was one of only five effective interventions for older adults implemented at scale [30–34]. CHAMPS effectively increased PA [35] was implemented and disseminated at an organizational level, and delivered through a variety of community organizations [35, 36]. Thus, we adopted core elements of CHAMPS for CTM (e.g. a choice-based, telephone assisted approach).

Our implementation approach aimed to reach older adults across settings. Thus, we incorporated knowledge translation principles and adopted a ‘framework for impact’ informed by a social ecological approach and participatory action research methods [20]. Specifically, we hosted a series of three to five meetings with delivery partners to facilitate input and to continually feedback the intervention design and implementation approach for further comment. Discussions focused on acceptability of the intervention to older adults, adaptability of the intervention to context and population (to assess scalability) and feasibility of implementation of CTM by Activity Coaches. Finally, the project team served as ‘experts’ (synthesis and translation system) to provide relevant evidence related to implementation frameworks and processes. Through these discussions, we adapted CHAMPS by adding more group interactions for Choose to Move to support social connectedness and co-created our phased and tailored approach to implementation of CTM at scale.

Specifically, CTM was designed to build community capacity that supports awareness of and access to local health promoting opportunities for older adults, as a means to enhance PA, mobility and feelings of social connectedness. CTM is a 6-month, choice-based health promotion intervention with a 3-month active phase and a 3-month maintenance phase.

CTM delivery partners spanned four levels of implementation as per the socioecological model of health promotion that recognizes multifaceted and interactive effects of person and environment on behaviours [37–39]. First, leads of provincial organizations (1 each at DP1 and DP2) served to liaise with the project team and to oversee operations at affiliate organizations. Second, recreation managers and recreation coordinators were responsible for participant recruitment, provision of infrastructure for meetings and programs (as needed) and logistic support to Activity Coaches. Finally, Activity Coaches interacted directly with older adult participants to deliver the CTM intervention.

Potential participants expressed interest in CTM to delivery partner organization staff who screened older adults for eligibility. Delivery partner staff also paired eligible participants with an Activity Coach. Activity Coaches were trained fitness leaders (DP1) or kinesiologists (DP2) hired by provincial partner organizations. The project team staff provided in-person 1-day content and process specific training for Activity Coaches. Technical assistance from the project team included on-going individual and group telephone consultations with Activity Coaches and consultations and feedback with recreation managers and recreation coordinators at every delivery site.

The CTM intervention was comprised of three core elements. First, a 60-min one-on-one consultation with Activity Coaches. During the one-on-one consultation Activity Coaches; i) assisted participants to set goals and create a personalized PA Action Plan tailored to participants’ interests, abilities, income and access to existing community-based PA resources, ii) offered guidelines on exercise progression, safety and addressed anticipated barriers to participation, and iii) encouraged participants to choose accessible community resources (e.g., walking clubs, recreation centre programs) or simple activities (e.g. walking, gardening) to sustain PA behaviour changes. Second, participants attended four 60-min Motivational Group Meetings (1x in months 1–2; 2x in month 3) to connect socially with other participants (max 12/group) and with their Activity Coach. Group sessions included presentations and group discussions on lifestyle topics that support developing and sustaining a PA Action Plan (i.e., active travel; healthy weights; chronic disease; stress and anxiety). Finally, Activity Coaches called participants regularly by phone (15 min/call on average) to monitor progress, address challenges and modify the Action Plan as needed (3x in month 1; 2x in months 2 and 3; 1x in months 4–6). Thus, participants received a greater “dose” of Activity Coach support during the active phase (one-on-one meeting, 7 telephone calls, 4 group meetings) compared to the maintenance phase (3 telephone calls). There was no cost for CTM as it was supported through a government grant.

Throughout the implementation process, the project team served as the prevention support system as per the Durlak and DuPre framework [27], and in this role adopted a variety of implementation strategies. These included developing strong community partnerships; design of an implementation blueprint; participation in ongoing meetings, interviews and focus groups; provision of program materials and tools; centralized oversight, training and technical assistance; convening advisory committees and a staged implementation scale-up approach. More details about these strategies are presented in Additional file 1.

### Measurements

We collected survey-based data from all participants (*n* = 458; province-wide assessment) and conducted more intensive evaluation in a subset of participants (*n* = 209; comprehensive assessment) enrolled in the 23 CTM programs in proximity to Greater Vancouver.

### Province-wide assessment

#### Demographic characteristics

All participants were asked to complete a baseline demographic survey including age category (60–74, ≥75), sex (male, female), self-reported height and weight, educational attainment (secondary school or less, at least some trade/technical school or college, at least some university), ethnicity (Asian, white, other), number of chronic diseases (0, 1, ≥2), self-rated health (very poor, poor, fair, good, excellent), self-efficacy for increasing PA (1 item) and accessing recreation centre services (1 item) (not at all, slightly, moderately, quite or very confident), and social support for PA received from family/friends (2 items).

#### Physical activity and mobility

At each measurement time point participants self-reported PA (1 item: number of days/week ≥30 min PA in the past week, [40] and capacity for mobility as no/any difficulty walking 400 m or climbing one flight of stairs (1 item) [41].

#### Social connectedness, loneliness and happiness

At each measurement time point, participants responded to three items to assess social connectedness. A sample item included “how many times in the past month have you said hello to a neighbour?”. The response options ranged from 0 = never to 5 = five or more times per week. Responses were used to calculate a summary score from 0 to 15; higher scores indicated less social exclusion [42]). Participants also completed items related to loneliness (3 items) [43] and global subjective happiness (4 items) [44]. A sample item for loneliness was, “how much of time do you feel left out” (often, some of the time, hardly). A summary score was calculated by totalling the three items (range 3–9); high scores indicated higher levels of loneliness. A sample item for happiness included “in general, I consider myself…” (1 = not a very happy person, 7 = a very happy person). A mean score was calculated across items (range 1–7); higher scores indicated greater happiness. Participants completed questionnaires at Motivational Group Meetings (0, 3 months) or at home. Questionnaires were returned via mail if participants missed a group meeting.

### Comprehensive subset assessment

#### Physical activity

Participants completed the 41-item CHAMPS questionnaire to assess weekly frequency and duration of a variety of physical activities typically undertaken by older adults [45]. We used the CHAMPS scoring algorithms to calculate energy expenditure (kcal/week) and frequency (times/week) for all exercise-related activities and moderate-intensity (≥3 METs) exercise-related activities.

#### Capacity for mobility and grip strength

We conducted a standardized mobility test using the Short Physical Performance Battery (SPPB) which evaluates standing balance, walking speed and timed chair stands [5] (summary score, range 0–12). We also assessed participant’s grip strength using a handheld dynamometer (Jamar Plus, Patterson Medical) to the nearest 0.1 kg. Participants performed two trials per side in a seated position with the arm supported at 90° and hand in a neutral position [46]. We combined the best trial from each side and report the sum as total grip strength. Assessments for the subset were completed at delivery partner facilities at 0, 3 and 6 months. At baseline we measured height to the nearest 0.1 cm using a portable stadiometer (Seca Model 214, Hanover MD, USA) and weight to the nearest 0.1 kg using a portable electronic scale (Seca model 840, Hanover MD, USA); we used the mean of two measures for analyses.

#### Reach and dose received

Throughout the intervention period, Activity Coaches used attendance logs to record in real-time participant’s attendance at group meetings (0–4 meetings; scored as 0–4) and the number of check-ins completed up to the end of the 4th group meeting (0, 1–2, 3–4, 5–6, 7; scored as 0–4). At the end of the active phase (3 months), Activity Coaches used attendance logs to complete a summary survey for each participant as a measure of reach [47]. Participants also responded to a single item regarding their satisfaction with CTM (dose received) [47].

### Statistical analysis

We performed all analyses using Stata v13.1 (StataCorp, College Station, TX USA). We compared participants’ socio-demographic characteristics at baseline by age, sex, delivery partner, subset and non-subset participants, and those who completed or did not complete the evaluation. To do so we used Chi-squared or Fisher’s exact test for categorical variables (sex, age category, ethnicity, education, chronic conditions, mobility limitations, subset participation) and unpaired t-tests for continuous variables (BMI).

We used general linear mixed effects models to describe the change in continuous variables during the intervention. For each continuous variable we fit a linear mixed model with time (0, 3, 6 months) as a categorical predictor. We first fit an empty means random intercept model and tested whether random slopes improved model fit using likelihood ratio tests. In Model 1 we included sex and age category as fixed effects. Model 2 included additional covariates: delivery partner, baseline mobility limitation, number of chronic conditions, education, and BMI. In both models we added fixed effects sequentially and tested interactions with time after the addition of each fixed effect. With the exception of an age*time interaction, interactions were only retained in the model if the Wald test [48] was significant (*p* < 0.05). We assessed model fit graphically using residual plots. Adjusted values were calculated at each time point using the margins command in Stata with a Bonferroni adjustment to account for multiple comparisons between and within age groups. Differences in the proportion of younger and older participants with some or significant mobility limitations was assessed at each time point using Chi-squared tests with Bonferroni adjustment for multiple comparisons (significance at: 0.05/3 = 0.017). We also used Chi-squared tests to assess differences in the proportion of participants with mobility limitations over time (0–3 months and 0–6 months) within each age group. Again we used a Bonferroni adjustment to account for multiple comparisons (significance at: 0.05/2 = 0.025). Finally, we assessed age- and sex-related differences in program dose using Wilcoxon rank sum tests and assessed the relationship between program reach and outcomes via Spearman rank correlations.

## Results

### Participants

Overall, most participants were women (77%), white (86%) and were living with one or more chronic conditions (overall: 87%, DP1: 99%; DP2: 81%) (Table 1). The most prevalent chronic condition was arthritis (50%) followed by hypertension (31%). Most participants (79%) reported having a friend or family member who encouraged them to be physically active. Fewer participants (53%) reported having someone who actively participated in PA with them.

**Table 1:** Baseline participant socio-demographic characteristics across the province. Values are n (%) or mean (SD)

	Total *n* = 56 programs *n* = 26 centres
Participants, n (men/women)	458 (105/353)
% (men)	23%
Age category, n (%)	
60–74 years	323 (71)
≥ 75 years	135 (29)
BMI, kg/m^2^	
Men	28.9 (4.5)
Women	29.5 (7.2)
Ethnicity, n (%)	
White	394 (86)
Asian	35 (8)
Other	29 (6)
Educational attainment, n (%)	
Secondary or less	113 (25)
Some trade, technical school or college	151 (33)
Some university	194 (42)
Chronic Conditions, n (%)	
0	61 (13)
1	185 (40)
≥ 2	212 (46)
Mobility limitations (walk or stair), n (%)	
Yes	196 (43)
No	262 (57)
Comprehensive cohort participants, n (%)	
Yes	209 (46)
No	249 (54)
Self-rated health, n (%) ^a^	
Very poor, poor or fair for age	206 (45)
Good or excellent for age	248 (55)
Self-efficacy for increasing PA, n (%) ^a^	
Not at all, slightly or moderately confident	206 (45)
Quite or very confident	248 (54)
Self-efficacy for rec centre access, n (%) ^b^	
Not at all, slightly or moderately confident	127 (34)
Quite or very confident	247 (66)

Note: Percentage values may not add exactly to 100 due to rounding

^a^total *n* = 454 (4 missing values)

^b^total *n* = 374 (84 missing values)

There were no significant differences in age, education, number of chronic conditions, participation in the subset, self-rated health or self-efficacy for increasing PA between men and women; however, women had more self-reported mobility limitations at baseline than did men (*p* = 0.02). The proportion of white participants (78% vs. 92%; *p* < 0.001) and proportion who rated their health as good or excellent for their age (49% vs. 59%, p = 0.02) was lower, while educational attainment was higher (48% vs 38% with at least some university; *p* = 0.03) in the comprehensive assessment subset compared with those who only completed the province-wide assessment. There were no differences in any other socio-demographic characteristic by subset status.

Overall 50 participants (11%) discontinued the program. The most common reasons given were, not applicable to them (*n* = 19), scheduling conflicts (*n* = 7) or family complications (*n* = 6). There were no significant differences in discontinuation by sex or age, but discontinuation was higher among DP2 vs DP1 participants (15% vs 5%, *p* < 0.001), by educational attainment (21 and 13% vs. 4% for secondary or less, at least some trade/technical/college and at least some university respectively, *p* < 0.01) and by mobility limitation at baseline (15% vs. 1% for no limitation vs. some limitation, *p* < 0.001) and comprehensive assessment subset status (15% vs. 6% for non-subset and subset respectively, *p* = 0.001).

### Province-wide assessment

#### Physical activity

We present PA outcomes for the whole sample (province-wide assessment) in Table 2. Results were similar for minimally and fully adjusted models, thus we focus on the fully adjusted model here. At baseline, participants aged ≥75 years were more physically active (+ 0.5 days/week; *p* = 0.02) than participants aged 60–74 yr. PA increased significantly during the active intervention phase (baseline to 3 months) in both younger (+ 1.6 days/week; *p* < 0.001) and older (+ 1.0 days/week; p < 0.001) participants. However, the increase was sustained at 6 months in younger participants only, who remained significantly more active than at baseline (+ 1.4 days/week; p < 0.001).

**Table 2:** Outcome measures by time point and age category in the provincial assessment (*n* = 458)

		Model 1				Model 2			
		60–74 y (*n* = 323)	≥ 75 y (*n* = 135)	p-value (60–74 y)	*p*-value (≥ 75 y)	60–74 y (n = 323)	≥ 75 y (*n* = 135)	p-value (60–74 y)	p-value (≥ 75 y)
				0–3 mo.	0–3 mo.			0–3 mo.	0–3 mo.
	Month			0–6 mo.	0–6 mo.			0–6 mo.	0–6 mo.
Physical activity	0	2.1 (1.9–2.4)	2.7 (2.3–3.0) *			2.1 (1.9–2.3)	2.7 (2.3–3.0) *		
(# days/week > 30 min)	3	3.8 (3.5–4.0)	3.6 (3.3–4.0)	**< 0.001**	**< 0.001**	3.7 (3.5–4.0)	3.6 (3.3–4.0)	**< 0.001**	**< 0.001**
	6	3.5 (3.3–3.7)	3.1 (2.7–3.4)	**< 0.001**	0.155	3.5 (3.3–3.7)	3.0 (2.6–3.4)	**< 0.001**	0.264
Social Exclusion	0	11.2 (10.9–11.5)	12.6 (12.2–13.1) *			11.1 (10.8–11.4)	12.8 (12.3–13.3) *		
(score, 0–15)	3	11.8 (11.4–12.1)	12.6 (12.0–13.1) *	**< 0.001**	> 0.99	11.7 (11.3–12.0)	12.7 (12.2–13.2) *	**< 0.001**	> 0.99
	6	11.6 (11.3–12.0)	12.5 (12.0–13.0) *	**0.018**	> 0.99	11.5 (11.2–11.9)	12.6 (12.1–13.1) *	**0.019**	> 0.99
Loneliness	0	4.7 (4.5–4.9)	4.1 (3.9–4.4) *			4.7 (4.5–4.9)	4.2 (3.9–4.5) *		
(score, 3–9)	3	4.3 (4.1–4.5)	3.7 (3.4–4.0) *	**< 0.001**	**< 0.001**	4.3 (4.1–4.5)	3.7 (3.5–4.0) *	**< 0.001**	**< 0.001**
	6	4.4 (4.2–4.6)	3.8 (3.5–4.1) *	**< 0.001**	**0.014**	4.4 (4.2–4.6)	3.8 (3.6–4.1) *	**< 0.001**	**0.010**
Sitting time	0	8.4 (8.0–8.7)	8.4 (7.8–8.9)			8.3 (7.9–8.6)	8.5 (8.0–9.1)		
(hours/day)	3	7.8 (7.5–8.2)	8.1 (7.5–8.7)	**0.02**	> 0.99	7.8 (7.4–8.2)	8.3 (7.7–8.8)	**0.02**	> 0.99
	6	7.9 (7.5–8.3)	7.2 (6.6–7.8)	0.06	**0.001**	7.9 (7.5–8.3)	7.4 (6.8–7.9)	0.08	**0.001**
Happiness	0	5.1 (4.9–5.2)	5.4 (5.2–5.6)*			5.1 (4.9–5.2)	5.4 (5.2–5.6)*		
(score, 1–7)	3	5.3 (5.1–5.4)	5.6 (5.4–5.8)*	**< 0.001**	**< 0.001**	5.3 (5.1–5.4)	5.6 (5.4–5.8)*	**< 0.001**	**< 0.001**
	6	5.2 (5.1–5.4)	5.6 (5.4–5.8)*	**0.001**	**0.001**	5.2 (5.1–5.4)	5.6 (5.4–5.8)*	**0.003**	**0.003**

Values are mean (95% CI)

Model 1 adjusted for age and gender

Model 2 additionally adjusted for delivery organization, baseline mobility, number of chronic conditions, education, and BMI (self-reported)

^*^Significant difference between age groups at specified time point

Significant differences between time points bolded

Note: Physical activity as measured by the single item questionnaire. For social exclusion, higher scores indicate less social exclusion. For loneliness, higher scores indicate greater loneliness. For happiness, higher scores indicate greater happiness. Sitting time censored to a max of 16.5 h/day (26 participants affected)

There were no significant interactions with sex, delivery partner, baseline mobility limitation, or educational attainment in the fully adjusted model. Across all time points men were more active than women (+ 0.6 days/week, *p* = 0.001). There were no differences in PA by baseline mobility limitation or by educational attainment. There was a significant interaction with number of chronic conditions; at baseline, participants with no chronic conditions were more physically active than those with one (+ 0.7 days/week; *p* = 0.015) or two or more (+ 1.0 d/week; p = 0.001). However, there were no differences by number of chronic conditions at 3 or 6 months. Model residuals were normally distributed but heteroscedastic indicating a potential problem with model fit (likely due to the interval and constrained nature of the data). Results were similar when both interval and censored (Tobit) regression (with robust standard errors to account for longitudinal data structure) were used.

#### Mobility

At baseline 43% of participants reported some mobility limitation, more amongst older participants (50%, compared to 40% among the younger group, *p* = 0.034). In younger participants, mobility limitations were significantly decreased at both 3 (25% vs. 40%; *p* < 0.001) and 6 (28% vs. 40%; *p* = 0.002) months compared to baseline. In older participants, mobility limitations were significantly decreased at 3 (36% vs. 50%; *p* = 0.02) but not 6 (39% vs. 50%; *p* = 0.07) months compared to baseline.

#### Social connectedness

Social exclusion scores by age group are shown in Table 2. In the fully adjusted model, older participants reported less social exclusion than younger participants at all time points (+ 1.0–1.7; *p* < 0.005 for all). In younger participants, social exclusion decreased from baseline to 3 (+ 0.6; *p* < 0.001) and 6 months (+ 0.4; p = 0.02). There was no change in social exclusion over time in older participants. There were significant interactions between social exclusion and sex and education. In general, men reported more social exclusion than did women (baseline: − 0.6, *p* = 0.05; 3 months: − 1.3, *p* < 0.001; 6 months: − 1.2, p < 0.001), and social exclusion was greater amongst those with least educational attainment.

#### Loneliness and happiness

Loneliness scores by age group are shown in Table 2. Across all time points older participants reported less loneliness than younger participants (0.5–0.6, *p* = 0.004–0.01). Loneliness decreased significantly during the active intervention phase (baseline to 3 months) in both younger (− 0.4; p < 0.001) and older (− 0.5; p < 0.001) participants. Loneliness remained lower at 6 months compared to baseline in both younger (− 0.3; *p* < 0.001) and older (− 0.4; *p* = 0.01) participants. There were differences in loneliness by sex; men reported less loneliness than women at baseline (− 0.5, *p* = 0.009) but not at 3 (− 0.4, *p* = 0.051) or 6 (− 0.1, *p* = 0.6) months. Happiness scores by age group are shown in Table 2. Older participants reported greater happiness than younger participants at all time points (+ 0.3; *p* = 0.007). There were no differences in happiness by sex or educational attainment.

### Comprehensive assessment

#### Physical activity (CHAMPS)

We present PA as exercise related energy-expenditure in the comprehensive sample by age group (Table 3). In the fully adjusted model there were no significant differences in energy expenditure between age groups at any time point (− 548 to + 650 kcal/week; *p* = 0.3–0.99). In younger participants weekly energy expenditure increased from baseline to 3 (+ 1149 kcal/week; *p* < 0.001) and 6 (+ 690 kcal/week; p < =0.005) months. Weekly energy expenditure did not change significantly in older participants (− 50 kcal/week and + 74 kcal/week at 3 and 6 months compared to baseline, respectively; *p* > 0.99 for both).

**Table 3:** Outcome measures by time point and age category in the comprehensive assessment subset

		Model 1				Model 2			
		60–74 y (*n* = 153)	≥ 75 y (*n* = 56)	*p*-value (60–74 y)	*p*-value (≥ 75 y)	60–74 y (*n* = 153)	≥ 75 y (*n* = 56)	*p*-value (60–74 y)	*p*-value (≥ 75 y)
				0–3 mo.	0–3 mo.			0–3 mo.	0–3 mo.
	Month			0–6 mo.	0–6 mo.			0–6 mo.	0–6 mo.
Physical activity	0	2528 (2197–2858)	2807 (2263–3352)			2451 (2126–2776)	2999 (2443–3556)		
(kcal/week, all exercise)	3	3656 (3272–4040)	2799 (2186–3412)	**< 0.001**	> 0.99	3600 (3219–3981)	2950 (2316–3584)	**< 0.001**	> 0.99
	6	3217 (2790–3643)	2877 (2179–3575)	**0.005**	> 0.99	3141 (2712–3570)	3073 (2355–3791)	**0.005**	> 0.99
Physical activity	0	1117 (895–1339)	1291 (924–1658)			1083 (864–1301)	1411 (1037–1785)		
(kcal/week, moderate exercise)	3	1813 (1548–2078)	1374 (951–1797)	**< 0.001**	> 0.99	1771 (1509–2032)	1531 (1098–1964)	**< 0.001**	> 0.99
	6	1568 (1267–1869)	1526 (1033–2019)	**0.02**	> 0.99	1517 (1217–1817)	1654 (1153–2155)	**0.02**	> 0.99
Physical activity	0	14.0 (12.3–15.6)	18.9 (16.1–21.6)*			14.1 (12.4–15.7)	18.9 (16.1–21.8)*		
(frequency/week, all exercise)	3	19.1 (17.4–20.9)	20.5 (17.7–23.3)	**< 0.001**	> 0.99	19.1 (17.4–20.9)	20.8 (17.9–23.8)	**< 0.001**	0.6
	6	19.0 (17.3–20.7)	19.1 (16.3–22.0)	**< 0.001**	> 0.99	19.1 (17.4–20.9)	19.2 (16.3–22.2)	**< 0.001**	> 0.99
Physical activity	0	4.3 (3.4–5.2)	6.0 (4.4–7.6)			4.5 (3.5–5.4)	6.2 (4.6–7.9)		
(frequency/week, moderate exercise)	3	7.4 (6.4–8.4)	7.6 (6.0–9.2)	**< 0.001**	0.2	7.5 (6.5–8.5)	7.6 (5.9–9.3)	**< 0.001**	0.3
	6	6.7 (5.7–7.7)	6.8 (5.2–8.4)	**< 0.001**	0.9	6.8 (5.8–7.9)	6.9 (5.2–8.6)	**< 0.001**	> 0.99
Mobility	0	10.1 (9.7–10.5)	9.2 (8.5–9.8) *			10.2 (9.8–10.5)	9.4 (8.8–10.0)		
(SPPB score, 1–12)	3	10.5 (10.1–10.9)	9.4 (8.8–10.1) *	**0.002**	0.4	10.5 (10.2–10.9)	9.8 (9.2–10.4)	**0.004**	0.1
	6	10.5 (10.1–10.9)	9.9 (9.2–10.5)	**0.009**	**0.005**	10.6 (10.2–10.9)	10.1 (9.5–10.8)	**0.02**	**0.007**
Grip Strength	0	51.7 (49.7–53.7)	43.4 (40.1–46.6) *			51.2 (49.2–53.3)	43.3 (39.8–46.7) *		
(kg)	3	53.7 (51.8–55.7)	44.0 (40.8–47.2) *	**0.001**	> 0.99	53.3 (51.3–55.3)	44.0 (40.6–47.4) *	**0.001**	> 0.99
	6	54.0 (52.0–56.0)	46.3 (43.1–49.6) *	**0.003**	**0.02**	53.6 (51.5–55.7)	46.1 (42.6–49.5) *	**0.003**	**0.046**

Values are mean (95% CI)

Model 1 adjusted for age and gender

Model 2 additionally adjusted for delivery organization, baseline mobility, number of chronic conditions, education, and BMI (measured)

^*^ Significant difference between age groups at specified time point

Significant differences between time points bolded

Note: Physical activity as measured by the CHAMPS physical activity questionnaire; SPPB, short performance physical battery

Across all time points energy expenditure was greater in men compared with women (+ 999 kcal/week, p < 0.001) and in those with at least some university education compared with at least some trade/technical/college education (+ 614 kcal/week; *p* = 0.03). At baseline, participants with a mobility limitation had lower energy expenditure than those with no mobility limitation (− 636 kcal/week; p = 0.03). However, there were no between group differences at 3 or 6 months. Participants with a mobility limitation at baseline increased energy expenditure over the course of the intervention; energy expenditure was increased at 3 (+ 1324 kcal/week; p < 0.001) and 6 (+ 733 kcal/week; p = 0.03) months compared to baseline. In contrast, those with no mobility limitation at baseline did not significantly increase energy expenditure at 3 (+ 448 kcal/week; *p* = 0.2) or 6 (+ 366 kcal/week; *p* = 0.4) months. Other components of the energy expenditure measures (frequency of all and of moderate activities) improved significantly for the younger age group at 3 and 6 months, but not for those aged over 75 years.

#### Mobility (SPPB)

Mobility outcomes by age group for the comprehensive cohort are shown in Table 3. In the fully adjusted model, there were no significant differences in SPPB score between age groups at any time point (0.4–0.7; *p* = 0.1–0.8). In younger participants, mobility increased significantly from baseline to 3 (+ 0.4; *p* = 0.004) and 6 (+ 0.4; *p* = 0.02) months. In older participants, mobility significantly increased at 6 months only, compared to baseline (+ 0.7; *p* = 0.007).

There were no differences in mobility by sex. Across all time points participants self-reporting a mobility limitation at baseline had lower SPPB scores than participants who reported no mobility limitations at baseline (− 1.6, *p* < 0.001).

#### Grip strength

Grip strength outcomes for the fully adjusted model are shown in Table 3. Across all time points, grip strength was greater in younger compared with older participants. Grip strength increased significantly during the active intervention phase (baseline to 3 months) in younger (+ 2.1 kg; *p* = 0.001) but not older (+ 0.7 kg; *p* > 0.99) participants. Compared to baseline, grip strength remained greater in younger participants (+ 2.4; *p* = 0.003) at 6 months. Grip strength was also greater at 6 months in older participants compared to baseline (+ 2.8; *p* = 0.046). However, examination of model residuals revealed one extreme outlier in the older participant group whose grip strength increased by almost 50 kg from baseline to 6 months. Re-running the model without this individual attenuated the findings such that the increase from 0 to 6 months was no longer statistically significant for older participants (+ 2.1; *p* = 0.1). Across all time points, men had greater grip strength than women (+ 31.0 kg; *p* < 0.001).

#### Reach and dose received

Overall, participants were highly engaged with the program. Of those who did not drop out (*n* = 408), 82% attended ≥75% of the group meetings and 95% completed ≥70% of the check-ins; 75.3% of the participants were satisfied with CTM. There were no age or sex-related differences in dose received, nor were there relationships between reach and most of our primary outcomes at baseline, change from baseline to 3 or 6 months (Table 4). Reach was negatively correlated with change in grip strength from baseline to 6 months, however upon further inspection, this change was driven by a few participants with low levels of reach and above average changes in grip strength.

**Table 4:** Spearman correlation between program dose and outcome measures

	Relationship between baseline value and program dose (Spearman’s rho)	Relationship between program dose and change baseline to 3 months	Relationship between program dose and change baseline to 6 months
Provincial assessment (n = 458)			
Physical activity (# days/week > 30 min)	0.05	0.06	0.06
Social Exclusion (score, 0–15)	0.06	−0.02	− 0.06
Loneliness (score, 3–9)	− 0.006	−0.01	0.01
Sitting time (hours/day)	−0.005	−0.07	− 0.004
Happiness (score, 1–7)	− 0.06	0.09	− 0.05
Comprehensive assessment subset (n = 209)			
Physical activity (kcal/week, all exercise)	0.15 (*p* = 0.03)*	−0.07	−0.06
Physical activity (kcal/week, moderate exercise)	0.14 (*p* = 0.049)*	−0.09	−0.06
Physical activity (frequency/week, all exercise)	0.09	0.03	0.12
Physical activity frequency/week, moderate exercise)	0.09	0.01	0.10
Mobility (SPPB score, 1–12)	−0.09	0.1	− 0.06
Grip Strength (kg)	0.05	−0.004	−0.19 (*p* = 0.01)*

*Significant correlation between program reach and outcome measure

## Discussion

A central tenet of successful public health promotion programs is a focus on populations rather than individuals [16]. These programs must be scalable which has been defined as “the ability to outgrow small, controlled research settings and replicate at scale in diverse real-world settings while retaining effectiveness” [49]. CTM is one of the first PA initiatives for older adults to be delivered at scale anywhere in Canada and our findings provide empirical evidence to support its scalability. In this iteration, CTM reached 534 seniors in 56 programs delivered across 26 sites in large and small urban regions of BC. Of these, we targeted and successfully enrolled low active older adults over age 60. This is a key population segment given the rapid growth of this demographic [2, 3], and the small proportion of older adults (13%) who engage in sufficient PA to promote their health [15]. Importantly, of 458 evaluated participants, 82% attended ≥75% of the group meetings and 95% completed ≥70% of check-ins.

Successfully enrolling this ‘elusive’ target population, high attendance rates and completion of selected CTM activities by participants, together highlight the essential role of community-based delivery partner organizations—a central element of scale up in diverse settings [25, 50]. The importance of partnerships was recognized in delivering scalable, sustainable PA to older adults in a Seattle based program [51]. Again, this is consistent with socioecological models that emphasize the need to engage multi-levels and multi-sectors [38, 52] through collaborative partnerships and ongoing stakeholder interaction [20]*.*

Specific to our study, we illustrated how with adequate central support, community-based delivery partners effectively implemented an evidence-based health. In addition, two key delivery partner organizations were selected based on their capacity to adapt, deliver and sustain a PA program for older adults and between them, maximize reach to older adults in communities across BC. Their network of fitness leaders/kinesiologists, recreation and other facilities and through them links to older adults, were key to recruitment success. Further, Yamey [53] supports that collaborating with non-governmental organizations (NGOs) as implementers, strong leadership, tailoring scale-up to the local situation, and integrating activities into existing program offerings as key to successful implementation at scale.

For the most part, participants in receipt of CTM from either delivery partner organization responded similarly to the intervention. However, there were slight differences by delivery partner in mobility score and energy expenditure at baseline. These differences might be explained by organizational reach to different geographic (DP1 – large urban centres with more ethnic diversity versus DP2 – small urban settings with less ethnic diversity) and demographic characteristics of older adult participants. Notably, to align with the mandate of their organization, DP1 stipulated the presence of at least one chronic condition as an eligibility criterion. Overall 86% of older adults presented with one or more chronic conditions; DP1 participants reported 99% versus 81% for DP2. These health differences of baseline could potentially limit capacity to participate fully in activity and underpin some of our findings. However, our study was not powered to differentiate outcomes by delivery partner.

In addition to successful replication of CTM in diverse settings, CTM positively influenced targeted health outcomes. PA and social connectedness improved (especially for the younger group aged 60–74) as did mental health (loneliness/happiness), grip strength and mobility in both younger and older groups. We observed a significant, short term (3 month) impact of CTM on PA (days/week over 30 min) of those aged 60–74 years (younger group) and those 75 years or older (older group). This increase during the active phase (4 group meetings; 7 check-ins) was maintained at 6 months in the younger group, despite diminished contact with participants (monthly check-ins) during the maintenance phase. This is a noteworthy finding given the need to minimize the cost of scaled up programs and should be pursued further in a purposely designed (cost effectiveness) study. Of those who completed the comprehensive, validated CHAMPS instrument [45], the younger group significantly increased their PA (energy expenditure and PA frequency) at both 3 and 6 months. The changes we observed in energy expenditure are similar in magnitude to those from the CHAMPS II randomized controlled trial and roughly equate to the addition of a 1 mile walk, 5 times per week – a substantial contribution to meeting PA guidelines [36]. These age-specific findings speak to the need for a flexible intervention that can be adapted for age and/or physical capacity. Active Choices, scaled-up as part of Active for Life, increased moderate-to-vigorous (+ 2.4 h/week) and total PA (+ 4.2 h/week) [54]. Importantly, despite greater diversity in ethnicity, socio-economic status and health conditions, the benefits we and others report are of similar magnitude to those reported for efficacy studies [31, 54].

At baseline, mobility limitation was reported by nearly half of CTM participants. Importantly, the proportion of participants who self-reported mobility limitations was reduced at 3 months for both age groups; benefits were maintained at 6 months for the younger group. For those who completed the comprehensive assessment, functional capacity (as measured by SPPB) was enhanced at 3 and 6 months for younger, and at 6 months for older participants. These findings are consistent with evidence from recent reviews that highlight the important role that PA plays to maintain functional capacity and reduce frailty [1, 55]. Importantly, low SPPB scores predict long term disability and/or institutionalization [56]. Although participants in CTM were generally not below critical thresholds, it is encouraging that participation in CTM enhanced this important marker of functional independence. It is also notable that PA gains (as measured by energy expenditure) were greater in those with self-reported mobility limitations at baseline, compared to those without mobility restrictions. This may reflect that CTM, a choice based, adaptable health promotion intervention, was able to accommodate differences in baseline mobility capacity.

CTM also enhanced social benefits for both age groups. Perceptions of social connectedness were reduced in the younger group, and loneliness decreased in both age groups. These benefits persisted across both time points compared to baseline, despite withdrawal of group sessions at three months. The association between perceived social isolation and health – including mortality [57], cardiovascular and mental health [58] – are well-established. Among the many health challenges older adults face as they age, the lack of social relationships is increasingly considered detrimental [59]. Although reports vary [60], older adults who are socially connected compared to those who are not, have better quality of life and a greater likelihood of survival [59–62]. Associations between PA and social connectedness are also well recognized [63–65], although relationships are likely to be bidirectional. Thus, the significant decreases in loneliness at 3 and 6 months we observed among younger and older CTM participants is encouraging. However, it is not possible to ascertain whether improvements were due to increased PA (possibly with others) or through social participation in CTM group sessions or to a greater sense of wellbeing overall.

That we observed no association between program reach and program outcomes might be explained by the relatively high attendance in group session and completion of check-ins across all sites. In a companion study of CTM participants, [data not shown] we used qualitative methods to discern factors that influenced implementation. Among five key elements, Activity Coaches were deemed by participants as crucial to continued participation. Similarly, delivery partners at all levels viewed activity coaches’ knowledge, enthusiasm and positivism as key to sustained participation. Activity coaches as paid hourly contractors, were motivated to deliver the intervention (*quantity*) as planned. As trained professionals they had the skills and experience *(quality)* to do so. Finally, empirical evidence to support a link between reach and program outcomes is weak compared with other factors that affect implementation such as dosage and fidelity [27].

Our evaluation of CTM has several limitations. First, we did not include a control group. However, as CTM was designed based on a program that previously demonstrated efficacy and effectiveness [35, 36] our intention was to examine translation to a new delivery context and at larger scale. Specifically we sought to determine whether the magnitude of program effects persisted, as it did when initially disseminated on a smaller scale [31, 54, 66]. Second, we used primarily self-report measures of PA and mobility as a means to reduce participant burden and to enhance feasibility of a province-wide evaluation. Self-report measures may be subject to social desirability bias. However, both PA questionnaires demonstrated acceptable validity with direct measures of PA [67, 68]. Third, there was limited ethnic diversity (86% white). This reflects demographics of British Columbia outside of metro Vancouver and may limit the generalizability of our findings. Finally, we do not know if the beneficial changes we observed are maintained after program completion. We are currently collecting follow up data (12 months after program completion i.e., 18 months from baseline) in order to address the question of maintenance. In this paper we report only two measures of implementation (i.e., program reach and dose received). In future, we consider it essential for all studies to very carefully examine other factors that influence implementation (e.g., dose delivered and quality) and where possible link implementation with outcomes.

Strengths of our evaluation include our cross-province reach, phased approach to scale up (allows for continuous feedback via Knowledge to Action cycle [69]) and adoption of a hybrid study design [28]. These features enable further dissemination to be assessed.

## Conclusions

CTM adopts central tenets of implementation and dissemination science that considers the complicated systems where we enact interventions to improve public health. In this iteration of CTM we describe scale up of an effective intervention delivered in 26 sites in large and small urban BC and demonstrate that partner-based model delivery can effectively enhance PA, mobility and dimensions of social well-being. Our overall approach reflects stages of implementation and evaluation, from formative evaluation in controlled settings through scale up in real world settings [70]. Based on a Knowledge to Action cycle [69] the model continues to be adapted based on results reported here and feedback from community partners across four levels (Activity Coaches, recreation coordinators, recreation managers and delivery partner organization leads) described in a companion study [25]. Scale up of CTM is ongoing and ultimately aims to reach thousands of geographically and culturally diverse older adults across BC [25].

Thus, the next phase of CTM will comprise vertical *scale up* (more sites same regions (less adaptation)) and *scale-out* (new sites, more remote and rural regions (more adaptation)) [71] as a means to disseminate CTM to more marginalized and less geographically accessible older adults [72]. This necessitates an expansion of the range of community partners and stakeholders to promote and deliver CTM. Ideally, this would be followed by institutionalization (and the related concept of “sustainability”) which will be based on CTM’s deliverability, affordability and effectiveness after broad dissemination across the province of BC [70].

## Additional file

Additional file 1: Choose to Move Implementation Strategies. Table includes an inventory of strategies for the implementation of Choose to Move. (DOCX 20 kb)

## Abbreviations

AART: Active Aging Research Team
BC: British Columbia
BMI: Body Mass Index
CHAMPS: Community Healthy Activities Model Program for Seniors
CTM: Choose to Move
PA: Physical Activity
SPPB: Short Physical Performance Battery

## References

[1] Bauman A, Merom D, Bull FC, Buchner DM, Fiatarone Singh MA (2016). Updating the evidence for physical activity: summative reviews of the epidemiological evidence, prevalence, and interventions to promote “active aging”. Gerontologist.

[2] Statistics Canada: Age and sex, and type of dwelling data: key results from the 2016 census. The Daily 2017. https://www150.statcan.gc.ca/n1/daily-quotidien/170503/dq170503a-eng.htm. Accessed 20 Sept 2017.

[3] Statistics Canada (2016). Canadian demographics at a glance.

[4] Heikkinen R. WHO ageing and health Programme: growing older - staying well: ageing and physical activity in everyday life. In: Institutional Repository for Information Sharing. Geneva: World Health Organization; 1998.

[5] Guralnik JM, Ferrucci L, Pieper CF, Leveille SG, Markides KS, Ostir GV, Studenski S, Berkman LF, Wallace RB (2000). Lower extremity function and subsequent disability: consistency across studies, predictive models, and value of gait speed alone compared with the short physical performance battery. J Gerontol A Biol Sci Med Sci.

[6] Dumurgier J, Elbaz A, Ducimetiere P, Tavernier B, Alperovitch A, Tzourio C (2009). Slow walking speed and cardiovascular death in well functioning older adults: prospective cohort study. BMJ.

[7] van Kan GA, Rolland Y, Andrieu S, Bauer J, Beauchet O, Bonnefoy M, Cesari M, Donini LM, Gillette-Guyonnet S, Inzitari M (2009). Gait speed at usual pace as a predictor of adverse outcomes in community-dwelling older people an international academy on nutrition and aging (IANA) task force. J Nutr Health Aging.

[8] Harwood RH, Conroy SP (2009). Slow walking speed in elderly people. BMJ.

[9] Newman AB, Simonsick EM, Naydeck BL, Boudreau RM, Kritchevsky SB, Nevitt MC, Pahor M, Satterfield S, Brach JS, Studenski SA (2006). Association of long-distance corridor walk performance with mortality, cardiovascular disease, mobility limitation, and disability. JAMA.

[10] Hirvensalo M, Rantanen T, Heikkinen E (2000). Mobility difficulties and physical activity as predictors of mortality and loss of independence in the community-living older population. J Am Geriatr Soc.

[11] Studenski S, Perera S, Patel K, Rosano C, Faulkner K, Inzitari M, Brach J, Chandler J, Cawthon P, Connor EB (2011). Gait speed and survival in older adults. JAMA.

[12] Potts MK (1997). Social support and depression among older adults living alone: the importance of friends within and outside of a retirement community. Soc Work.

[13] Foley DJ, Ostfeld AM, Branch LG, Wallace RB, McGloin J, Cornoni-Huntley JC (1992). The risk of nursing home admission in three communities. J Aging Health.

[14] Hallal PC, Andersen LB, Bull FC, Guthold R, Haskell W, Ekelund U (2012). Global physical activity levels: surveillance progress, pitfalls, and prospects. Lancet.

[15] Colley RC, Garriguet D, Janssen I, Craig CL, Clarke J, Tremblay MS (2011). Physical activity of Canadian adults: accelerometer results from the 2007 to 2009 Canadian health measures survey. Health Rep.

[16] Koplan JP, Bond TC, Merson MH, Reddy KS, Rodriguez MH, Sewankambo NK, Wasserheit JN (2009). Towards a common definition of global health. Lancet.

[17] Reis Rodrigo S, Salvo Deborah, Ogilvie David, Lambert Estelle V, Goenka Shifalika, Brownson Ross C (2016). Scaling up physical activity interventions worldwide: stepping up to larger and smarter approaches to get people moving. The Lancet.

[18] Milat AJ, Bauman AE, Redman S, Curac N (2011). Public health research outputs from efficacy to dissemination: a bibliometric analysis. BMC Public Health.

[19] World Health Organization. Bridging the “know–do” gap. In: Meeting on knowledge translation in Global Health. Geneva: World Health Organiation; 2006.

[20] Haggis C, Sims-Gould J, Winters M, Gutteridge K, McKay H (2013). Sustained impact of community-based physical activity interventions: key elements for success. BMC Public Health.

[21] Sanders EBN, Stappers PJ (2008). Co-creation and the new landscapes of design. CoDesign.

[22] Greenhalgh T, Jackson C, Shaw S, Janamian T (2016). Achieving research impact through co-creation in community-based health services: literature review and case study. The Milbank Quarterly.

[23] Leask Calum F, Sandlund Marlene, Skelton Dawn A, Chastin Sebastien FM (2017). Co-creating a tailored public health intervention to reduce older adults’ sedentary behaviour. Health Education Journal.

[24] Ministry of Health (2015). Active people, active places: British Columbia physical activity strategy. Healthy families BC.

[25] McKay HA, Sims-Gould J, Nettlefold L, Hoy CL, Bauman AE. Implementing and evaluating an older adult physical activity model at scale: framework for action. Transl J of ACSM. 2017;2(2):10–9.

[26] Wandersman A, Duffy J, Flaspohler P, Noonan R, Lubell K, Stillman L, Blachman M, Dunville R, Saul J (2008). Bridging the gap between prevention research and practice: the interactive systems framework for dissemination and implementation. Am J Community Psychol.

[27] Durlak JA, DuPre EP (2008). Implementation matters: a review of research on the influence of implementation on program outcomes and the factors affecting implementation. Am J Community Psychol.

[28] Curran GM, Bauer M, Mittman B, Pyne JM, Stetler C (2012). Effectiveness-implementation hybrid designs: combining elements of clinical effectiveness and implementation research to enhance public health impact. Med Care.

[29] Canadian Society for Exercise Physiology (2012). The physical activity readiness questionnaire for everyone.

[30] Harris T, Kerry SM, Limb ES, Furness C, Wahlich C, Victor CR, Iliffe S, Whincup PH, Ussher M, Ekelund U (2018). Physical activity levels in adults and older adults 3-4 years after pedometer-based walking interventions: long-term follow-up of participants from two randomised controlled trials in UK primary care. PLoS Med.

[31] Wilcox S, Dowda M, Leviton LC, Bartlett-Prescott J, Bazzarre T, Campbell-Voytal K, Carpenter RA, Castro CM, Dowdy D, Dunn AL (2008). Active for life: final results from the translation of two physical activity programs. Am J Prev Med.

[32] Phelan EA, Williams B, Snyder SJ, Fitts SS, LoGerfo JP (2006). A five state dissemination of a community-based disability prevention program for older adults. Clin Interv Aging.

[33] Seguin RA, Economos CD, Hyatt R, Palombo R, Reed PN, Nelson ME (2008). Design and national dissemination of the StrongWomen community strength training program. Prev Chronic Dis.

[34] Stewart AL, Gillis D, Grossman M, Castrillo M, Pruitt L, McLellan B, Sperber N (2006). Diffusing a research-based physical activity promotion program for seniors into diverse communities: CHAMPS III. Prev Chronic Dis.

[35] Stewart AL, Mills KM, Sepsis PG, King AC, McLellan BY, Roitz K, Ritter PL (1997). Evaluation of CHAMPS, a physical activity promotion program for older adults. Ann Behav Med.

[36] Stewart AL, Verboncoeur CJ, McLellan BY, Gillis DE, Rush S, Mills KM, King AC, Ritter P, Brown BW, Bortz WM (2001). Physical activity outcomes of CHAMPS II: a physical activity promotion program for older adults. J Gerontol A Biol Sci Med Sci.

[37] Golden SD, Earp JA (2012). Social ecological approaches to individuals and their contexts: twenty years of health education & behavior health promotion interventions. Health Educ Behav.

[38] Stokols Daniel (1996). Bridging the theoretical and applied facets of environmental psychology. American Psychologist.

[39] Bronfenbrenner U (1994). Ecological models of human development, vol. Vol. 3.

[40] Milton K, Bull FC, Bauman A (2011). Reliability and validity testing of a single-item physical activity measure. Br J Sports Med.

[41] Simonsick EM, Newman AB, Visser M, Goodpaster B, Kritchevsky SB, Rubin S, Nevitt MC, Harris TB, Health A, Body Composition S (2008). Mobility limitation in self-described well-functioning older adults: importance of endurance walk testing. J Gerontol A Biol Sci Med Sci.

[42] Veroff J, Kulka RA, Douvan E (1981). Mental health in America: patterns of help-seeking from 1957–1976.

[43] Hughes ME, Waite LJ, Hawkley LC, Cacioppo JT (2004). A short scale for measuring loneliness in large surveys: results from two population-based studies. Res Aging.

[44] Lyubomirsky S, Lepper HS (1999). A measure of subjective happiness: preliminary reliability and construct validation. Soc Indic Res.

[45] Stewart AL, Mills KM, King AC, Haskell WL, Gillis D, Ritter PL (2001). CHAMPS physical activity questionnaire for older adults: outcomes for interventions. Med Sci Sports Exerc.

[46] Roberts HC, Denison HJ, Martin HJ, Patel HP, Syddall H, Cooper C, Sayer AA (2011). A review of the measurement of grip strength in clinical and epidemiological studies: towards a standardised approach. Age Ageing.

[47] Saunders RP, Evans MH, Joshi P (2005). Developing a process-evaluation plan for assessing health promotion program implementation: a how-to guide. Health Promot Pract.

[48] Wald A (1945). Sequential tests of statistical hypotheses. Ann Math Statist.

[49] Milat AJ, King L, Bauman AE, Redman S (2012). The concept of scalability: increasing the scale and potential adoption of health promotion interventions into policy and practice. Health Promot Int.

[50] Hanson K, Ranson MK, Oliveira-Cruz V, Mills A (2003). Expanding access to priority health interventions: a framework for understanding the constraints to scaling-up. J of Int Dev.

[51] Cheadle A, Egger R, Logerfo JP, Schwartz S, Harris JR (2010). Promoting sustainable community change in support of older adult physical activity: evaluation findings from the Southeast Seattle senior physical activity network (SESPAN). J of Urban Health.

[52] King AC, Stokols D, Talen E, Brassington GS, Killingsworth R (2002). Theoretical approaches to the promotion of physical activity: forging a transdisciplinary paradigm. Am J Prev Med.

[53] Yamey G (2011). Scaling up global health interventions: a proposed framework for success. PLoS Med.

[54] Wilcox S, Dowda M, Griffin SF, Rheaume C, Ory MG, Leviton L, King AC, Dunn A, Buchner DM, Bazzarre T (2006). Results of the first year of active for life: translation of 2 evidence-based physical activity programs for older adults into community settings. Am J Public Health.

[55] Chase JD, Phillips LJ, Brown M (2017). Physical activity intervention effects on physical function among community-dwelling older adults: a systematic review and meta-analysis. J Aging Phys Act.

[56] Gawel J, Vengrow D, Collins J, Brown S, Buchanan A, Cook C (2013). The short physical performance battery as a predictor for long term disability or institutionalization in the community dwelling population aged 65 years old or older. Phys Therapy Rev.

[57] Holt-Lunstad J, Smith TB, Baker M, Harris T, Stephenson D (2015). Loneliness and social isolation as risk factors for mortality: a meta-analytic review. Perspect Psychol Sci.

[58] Leigh-Hunt N, Bagguley D, Bash K, Turner V, Turnbull S, Valtorta N, Caan W (2017). An overview of systematic reviews on the public health consequences of social isolation and loneliness. Public Health.

[59] World Health Organization (2015). World report on ageing and health.

[60] Poscia A, Stojanovic J, La Milia DI, Duplaga M, Grysztar M, Moscato U, Onder G, Collamati A, Ricciardi W, Magnavita N (2018). Interventions targeting loneliness and social isolation among the older people: an update systematic review. Exp Gerontol.

[61] Ellwardt L, van Tilburg T, Aartsen M, Wittek R, Steverink N (2015). Personal networks and mortality risk in older adults: a twenty-year longitudinal study. PLoS One.

[62] Tabue Teguo M, Simo-Tabue N, Stoykova R, Meillon C, Cogne M, Amieva H, Dartigues JF (2016). Feelings of loneliness and living alone as predictors of mortality in the elderly: the PAQUID study. Psychosom Med.

[63] Lindsay Smith G, Banting L, Eime R, O’Sullivan G, van Uffelen JGZ (2017). The association between social support and physical activity in older adults: a systematic review. Int J Behav Nutr Phys Act.

[64] Shvedko A, Whittaker AC, Thompson JL, Greig CA (2018). Physical activity interventions for treatment of social isolation, loneliness or low social support in older adults: a systematic review and meta-analysis of randomised controlled trials. Psych of Sport and Exerc.

[65] Stathi A, McKenna J, Fox K (2004). The experiences of older people participating in exercise referral schemes. J R Soc Promot Heal.

[66] Wilcox S, Dowda M, Wegley S, Ory MG (2009). Maintenance of change in the active-for-life initiative. Am J Prev Med.

[67] Milton K, Clemes S, Bull F (2013). Can a single question provide an accurate measure of physical activity?. Br J Sports Med.

[68] Harada ND, Chiu V, King AC, Stewart AL (2001). An evaluation of three self-report physical activity instruments for older adults. Med Sci Sports Exerc.

[69] Graham ID, Logan J, Harrison MB, Straus SE, Tetroe J, Caswell W, Robinson N (2006). Lost in knowledge translation: time for a map?. J Contin Educ Heal Prof.

[70] Bauman A, Nutbeam D (2014). Evaluation in a nutshell.

[71] Aarons GA, Sklar M, Mustanski B, Benbow N, Brown CH (2017). “Scaling-out” evidence-based interventions to new populations or new health care delivery systems. Implement Sci.

[72] Milat AJ, Newson R, King L, Rissel C, Wolfenden L, Bauman A, Redman S, Giffin M (2016). A guide to scaling up population health interventions. Public Health Res Pract.

[73] Harris PA, Taylor R, Thielke R, Payne J, Gonzalez N, Conde JG (2009). Research electronic data capture (REDCap)--a metadata-driven methodology and workflow process for providing translational research informatics support. J Biomed Inform.

