# Supplementary material for: Implementation of a co-designed physical activity program for older adults: positive impact when delivered at scale
Source: BMC Public Health. 2018 Nov 23;18:1289. doi: 10.1186/s12889-018-6210-2 (PMC6251145; doi:10.1186/s12889-018-6210-2)
Supplement: Supplementary file 1 — Choose to Move Implementation Strategies. Table includes an inventory of strategies for the implementation of Choose to Move. (DOCX 20 kb) [file 12889_2018_6210_MOESM1_ESM.docx]

Additional file 1

Choose to Move (CTM) Implementation Strategies

| CTM Implementation Strategies [1] | Descriptions |
| --- | --- |
| Conduct needs assessment (at provincial partner level) | Prior to CTM implementation, the CTM project team (project team)* conducted formative evaluation using semi-structured focus interviews to assess   1. Older adults’ acceptability of CTM 2. Delivery partners’ perceived adaptability of CTM to context and population 3. Activity coaches’ perceived feasibility of implementing CTM implementation of the intervention by Activity Coaches (to assess feasibility) and to identify and pilot evaluation tools and methods to assess effectiveness of CTM at scale. We collaborated with both delivery partners to adapt the program to their organizational context |
| Develop community partnership and  obtain formal commitments | The project team partnered with the two community organizations to deliver CTM at their affiliated facilities. Both organizations have signed contract agreement committing on program delivery. |
| Develop program materials and tools | The project team developed and provided the following materials for the delivery organization. These include materials for:   1. Program managers  - Recruitment materials - Descriptions of program coordinators and activity coaches hiring process and job descriptions  1. Program coordinators  - Outline of implementation and evaluation tasks  1. Activity coaches  - Presentation materials on health topics for motivational group meeting sessions - Tools to record participants attendance and responses during one-on-one Action Planning and telephone check-ins. |
| Centralized technical assistance | The project team functioned as the prevention support system to provide centralized technical assistance to the program coordinators, managers and activity coaches. |
| Conduct dynamic training | The project team provided a 1-day training for activity coaches. Training content included overview of CTM, motivational interviewing techniques, active listening skills. They were provided with skills demonstration, opportunities to practice the learned skills and ask questions. |
| Provide on-going consultation | The project team provided on-going telephone consultations to each delivery sites throughout the intervention period. The purpose of these phone calls was to identify and troubleshoot implementation issues. These included:   - Regular phone call (weekly to start, then monthly as Phase II progressed) with provincial coordinators - On-going email communications to provide additional support |
| Use advisory boards and workgroups | During CTM implementation, the project team formed two advisory committees that provide ongoing feedback we use to adapt the program as needed throughout the intervention period. Both advisory committees meet annually.   - The Community Advisory Committee comprises older adult participants, recreation coordinators, and activity coaches from partner organizations and members of the Active Aging Research (AART) team. This committee shares lessons learned during the implementation of CTM. - The Leadership Advisory Committee comprises leaders of delivery partner organizations and members of AART. This committee was the organizational lens we used to monitor the implementation of CTM in collaboration with partner organizations and to assess the need for further adaptation of CTM to meet the specific needs and capacity of delivery organizations before scale-up. Both advisory committees meet annually. |
| Stage implementation scale up | CTM was first piloted in DP2 (8 communities) in Phase I before a larger scale roll out in DP1 and DP2 in Phase II (48 communities). This pilot provided opportunity for delivery partners to provide feedback on the feasibility of CTM implementation and identify barriers and facilitators of implementation. This feedback was then used to refine the intervention and implementation plan in Phase II. |

* The Choose to Move project team (project team) was convened by the Active Aging Research Team to support delivery of CTM. Specifically, the project team comprised of two principal investigators (HM, JSG), international research collaborators, a program manager and several research assistants to support day-to-day operation and program evaluation.

**Reference**

1. Powell BJ, Waltz TJ, Chinman MJ, Damschroder LJ, Smith JL, Matthieu MM, Proctor EK, Kirchner JE: **A refined compilation of implementation strategies: results from the Expert Recommendations for Implementing Change (ERIC) project**. *Implementation Science* 2015, **10**(1):21.
